# Latent infections, coronavirus disease 2019 and psychiatric disorders: The friend of my enemy

**DOI:** 10.1002/ctd2.141

**Published:** 2022-10-13

**Authors:** Mirza Ali Mofazzal Jahromi, Mina Sefidfard, Ali Taghipour, Abazar Roustazadeh, Sara Matin, Hamed Mir, Milad Badri, Fares Bahrami, Amir Abdoli

**Affiliations:** ^1^ Zoonoses Research Center Jahrom University of Medical Sciences Jahrom Iran; ^2^ Department of Immunology School of Medicine Jahrom University of Medical Sciences Jahrom Iran; ^3^ Department of Advanced Medical Sciences & Technologies Jahrom University of Medical Sciences Jahrom Iran; ^4^ Department of Psychiatry Jahrom University of Medical Sciences Jahrom Iran; ^5^ Research Center for Noncommunicable Diseases Jahrom University of Medical Sciences Jahrom Iran; ^6^ Department of Parasitology and Mycology Jahrom University of Medical Sciences Jahrom Iran; ^7^ Department of Biochemistry and Nutrition Jahrom University of Medical Sciences Jahrom Iran; ^8^ Department of Pediatrics Jahrom Iran; ^9^ Medical Microbiology Research Center Qazvin University of Medical Sciences Qazvin Iran; ^10^ Kurdistan University of Medical Sciences Kurdistan Iran

**Keywords:** COVID‐19, latent infections, psychiatric disorders, SARS‐CoV‐2

## Abstract

Recent reports revealed an increased rate of hospitalization and mortality of coronavirus disease 2019 (COVID‐19) among patients with psychiatric disorders. On the other hand, there is a link between latent infections, including *Toxoplasma gondii*, herpes simplex virus type 1 (HSV‐1) and cytomegalovirus (CMV) with psychiatric disorders. We individually assessed data regarding 1) the mortality rate of COVID‐19 among individuals with psychiatric disorders; 2) the association of latent infections in COVID‐19 patients and 3) the association between latent infections and psychiatric disorders. We developed the hypothesis that latent infection could increase the risk of severe COVID‐19 among patients with psychiatric disorders. Cumulative evidence proposed that infection with toxoplasmosis, CMV and HSV‐1 could increase the risk of severe acute respiratory syndrome coronavirus 2 (SARS‐Co‐V2) infections among patients with psychiatric disorders probably by induction of hyperinflammatory conditions. These infections are also associated with hyperinflammation and T cell exhaustion, which has also been observed in both schizophrenia and COVID‐19. This hypothesis provides new insights into the role of latent infections in increasing the mortality rates of COVID‐19 among individuals with psychiatric disorders. Strategies for screening, early diagnosis and treatment of these infections could be recommended for COVID‐19 patients with a background of psychiatric disorders.

## 1 BACKGROUND

### 1.1 Opportunistic infections increase mortality rates of patients with coronavirus disease 2019 (COVID‐19)

Immune disturbances, including hyperinflammation and cytokine storm syndrome (CSS), are key players in developing severe sequels of severe acute respiratory syndrome coronavirus 2 (SARS‐CoV‐2) infection, such as acute respiratory distress syndrome and multiorgan failure and death. Immunomodulatory therapies (by corticosteroids or other agents) have shown promising outcomes for the control of CSS among patients with COVID‐19 and decreased mortality rates.^1^ However, immunomodulatory therapy can increase the risk of reactivation of latent toxoplasmosis, cytomegalovirus (CMV) and herpes simplex virus type (HSV)‐infections.^1^ In this regard, several cases of severe and fatal toxoplasmosis, CMV and HSV‐1 infections have been reported among COVID‐19 patients, most of which received immunomodulatory therapies (Reviewed in).1 A higher incidence of CMV and HSV‐1 were reported among severe COVID‐19 cases.^2^ Latent CMV infection was associated with an increased risk of COVID‐19‐related hospitalization.^3^ Critically ill patients with severe COVID‐19 were reported to be at a higher risk for CMV and epstein‐barr virus (EBV) reactivation.^4^ Toxoplasmosis could be a risk factor for the severity of SARS‐CoV‐2 infection.^5^

### 1.2 Increased mortality rate of COVID‐19 among individuals with psychiatric disorders

The global COVID‐19 pandemic has presented a complicated challenge among patients with psychiatric disorders, while higher hospitalization and mortality rates have been reported among these patients.^6^, ^7^ The results of a recent meta‐analysis revealed that the mortality rate of COVID‐19 is 1.75‐time higher among patients with mental health disorders compared to infected patients without mental health disorders (odds ratio [OR] = 1.75^8^; *p* <  .05). As such, the COVID‐19 patients with severe mental health disorders had an increased mortality rate than patients without severe mental health disorders (OR: 2.26 [95% confidence interval [CI], 1.18–4.31])).^8^

### 1.3 Association between neurotropic infections and psychiatric disorders

Neurotropic infections (toxoplasmosis, CMV and HSV‐1) are highly prevalent infections among the general population, and their infections are usually asymptomatic (latent) in immunocompetent individuals; however, immunocompromised patients or pregnant women may have severe sequels with fatal outcome. Nevertheless, several neuropsychiatric disorders have been linked to these latent infections.^9^ Studies indicated the link between schizophrenia and psychiatric disorders with *Toxoplasma (T). gondii*, CMV and HSV‐1 infection.^9^ Hamdani et al.^10^ have shown significant associations between seropositivity to *T. gondii*, HSV‐1, HSV‐2, and CMV with lower cognitive functions among patients with schizophrenia and bipolar disorder.^10^ Shirts et al.^11^ found that seropositivity to HSV‐1 and CMV was significantly associated with impaired cognitive function among patients with schizophrenia disorder.^11^ Increased seropositivity to *T. gondii* infection was reported among treatment‐resistant schizophrenia patients.^12^ Holub et al.^13^ found that hospitalization of schizophrenia patients with *T. gondii* infection was increased during their last admission compared to *T. gondii*‐seronegative schizophrenia patients (*p* = 0.003; mean difference 32.9 days). As such, the *T. gondii* seropositive patients had higher levels in the Positive Subscale of Positive and Negative Symptom Scale (PANSS); as well, as the concentrations of anti‐*T. gondii* antibodies were negatively associated with the PANSS scores.^13^ The result of a meta‐analysis revealed that *T. gondii*‐IgG seropositivity was significantly increased among patients with bipolar disorder (OR 1.52, *p* = 0.02), schizophrenia (OR 1.81, *p* < 0.00001), addiction (OR 1.91, *p* < 0.00001) and OCD (OR 3.4, *p* < 0.001).^14^ Taken together, cumulative evidence revealed an association between these neurotropic infections and psychiatric disorders.

## 2 THE HYPOTHESIS/THEORY

It is a hypothesis that infection with toxoplasmosis, CMV, and HSV could increase the risk of severe COVID‐19 among patients with psychiatric disorders.

## 3 EVALUATION OF THE HYPOTHESIS/IDEA

Neurotropic infections like toxoplasmosis, CMV, and HSV augment inflammatory reactions.^9^ It is also proposed that co‐infection of these pathogens synergically augments the level of inflammatory reactions more than their single infection.^9^ Therefore, these infections could have an indirect role in increasing the mortality rate of COVID‐19, probably by induction of CSS. On the other hand, there is a strong connection between the severity of neuropsychiatric disorders with inflammatory immune responses.^9^ While, anti‐inflammatory therapies have been prescribed as an adjuvant in patients with major depressive disorder in a number of studies.^15^, ^16^ Anti‐inflammatory drugs have been widely used for the modulation of CCS in patients with severe COVID‐19.^1^ Hence, anti‐inflammatory therapy in schizophrenia and COVID‐19 could increase the risk of severe toxoplasmosis, CMV, or HSV infection.

**FIGURE 1:**
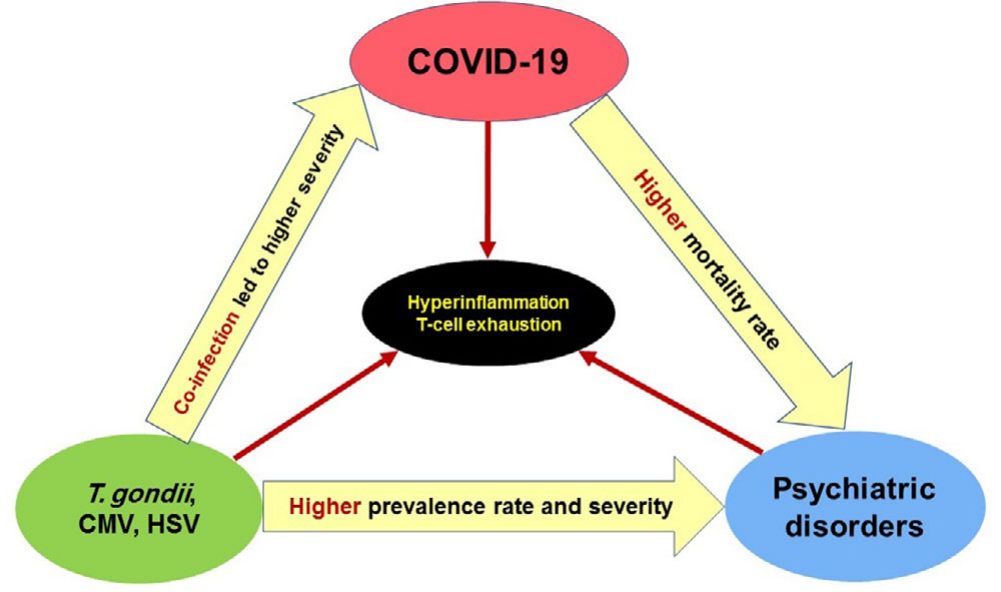
A hypothetical scheme linking COVID‐19, psychiatric disorders, and neurotropic infections

Recent reports revealed immune disturbances in COVID‐19 patients, including T cell exhaustion, and a marked increase of proinflammatory and inflammatory cytokines, such as interferon‐γ, tumour necrosis factor‐a, interleukin (IL)‐2 and IL‐17.^17^ T cell exhaustion has also linked to neurological manifestations of COVID‐19.^18^ Remarkably, there is also a strong link between *T. gondii*, CMV, and HSV‐1 infection with T cell (especially CD8 T cells) exhaustion.^19^, ^20^, ^21^ Indeed, the altered T cell function has been reported among patients with schizophrenia^22^, ^23^ and in animal models of mental disorders.^24^ Hence, these latent infections could have a risk factor for COVID‐19 severity, especially among schizophrenia patients. Figure 1 presents a hypothetical scheme linking COVID‐19, psychiatric disorders and neurotropic infections.

## 4 CONSEQUENCES OF THE HYPOTHESIS AND DISCUSSION

In conclusion, *T. gondii*, CMV, and HSV‐1 infections may have an indirect role in increasing the mortality rate of COVID‐19 among patients with psychiatric disorders. Co‐infection of these pathogens may also increase the mortality rate due to their synergistic effects. Screening, early diagnosis and treatment of these neurotropic infections might mitigate the severe sequel of COVID‐19 among patients with a background of psychiatric disorders.

## AUTHOR CONTRIBUTIONS

Dr. Abdoli conceived the idea. Dr. Mofazzal and Dr. Abdoli contributed to the preparation and collection of original literatures and figures and the writing and editing of manuscript. Dr. Sefidfard, Dr Taghipour, Dr. Roustazadeh, Dr. Matin, Dr. Mir, Dr. Badri and Dr. Bahrami were responsible for the structural designs, scientific quality and writing.

## FUNDING INFORMATION

Not applicable.

## CONFLICT OF INTEREST

The authors declare no conflict of interest. The paper was handled by editors and has undergone a rigorous peer‐review process. Dr. Amir Abdoli was not involved in the journal's review of/or decisions related to this manuscript.

## ETHICAL APPROVAL

Not applicable.

## Data Availability

Data sharing is not applicable to this article as no new data were created or analyzed in this study.
